# Complexity of Chloramine Decay Kinetics in Premise Plumbing

**DOI:** 10.1021/acsestwater.5c01339

**Published:** 2026-01-30

**Authors:** Tolulope O. Odimayomi, Darel C. Snead, Amy Pruden, Marc A. Edwards

**Affiliations:** † Via Department of Civil and Environmental Engineering, 1757 Virginia Tech, Blacksburg, Virginia 24061, United States

**Keywords:** Disinfectant Decay Kinetics, Nitrification, Temperature, N^th^ Order Model

## Abstract

Nitrification-driven chloramine decay kinetics have largely been unquantified in premise plumbing, which is particularly vulnerable to opportunistic pathogen growth. Here, we carried out complementary experiments in an at-scale premise plumbing rig with mature biofilms (>4 years age) with influent residuals of <0.2, 0.25, 0.5, 1.0, and 2.5 mg/L as Cl_2_ and sterile glass jars, with and without an inoculum containing nitrifying bacteria. Chloramine decay was complete after 8 h of stagnation in all PEX rig pipes (n = 16), tested over a range of diameters (1/4–3/4”) and flow rates (0.25–2.2 gpm), with decay rates increasing in situations with higher nitrification rates. The jar experiments revealed that chloramine actually persisted better at higher (37–39 °C) than lower (19–30 °C) temperatures, contrary to standard temperature-adjusted kinetic assumptions, presumably because nitrifiers are inhibited at higher temperatures. Contrary to assumptions made in conventional models, chloramine decay was only effectively modeled as first order in 8/24 cases in the rig experiment (R^2^ > 0.9). The best fit chloramine decay reaction order varied among the rig pipes from 0.88 to 2.74, depending on chloramine dose and exposure time, hydraulics, and modeling method.

## Graphical abstract

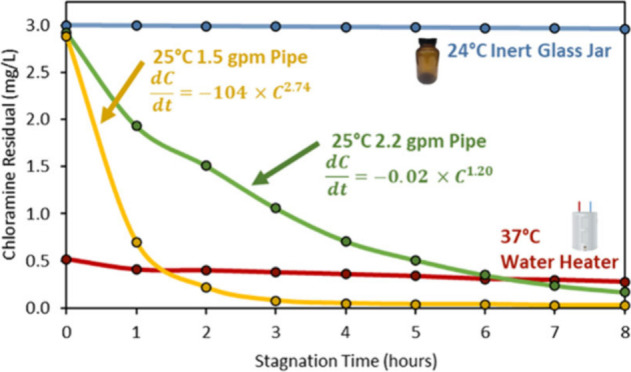

## Introduction

Drinking water distribution systems (DWDSs) must meet regulatory requirements for disinfection byproducts, control of microbial growth, and detectable disinfectant residuals.^1–5^ Many utilities throughout the world achieve this through the addition of chloramine as a secondary disinfectant residual. The ability to maintain effective chloramine residual can be influenced by decay reactions including (1) autodecomposition,^5^ (2) chemical reactions with organic matter and inorganic ions in the bulk water,^3,6–11^ (3) biological reactions, especially nitrification, typically driven by biofilm,^12–17^ and (4) corrosion reactions with the pipe wall.^18–21^ Such phenomena can be interactive and exacerbated in premise plumbing.^16,19,22^ In order to effectively maintain disinfection capacity throughout the drinking water system, an integrated understanding is needed of the range of conditions under which such phenomena dominate, independently or synergistically, the drive of chloramine decay.

Various experimental scales and designs are needed in order to differentiate the factors driving the chloramine decay kinetics. Autogenous and bulk water disinfectant decay reactions can be examined in inert containers. From such studies, it has been found that chloramine decay is determined by residual concentration, chlorine to ammonia nitrogen ratio, nitrite concentration and bromide concentration, whereas other constituents such as carbonate, phosphate, and soluble microbial products can catalyze decay.^5,10,23–25^ A generalized chloramine decay model of

1 $$\frac{dC}{dt}=-kC^{n}$$

can be used to fit data, in which *C* is chlorine concentration at time *t* (mg/L), *n* is the reaction order, and *k* is the chlorine reaction rate coefficient (variable units dependent on *n*). Bulk water decay in water distribution systems with large pipes is often assumed to be first order,^26–30^ but some studies empirically determined that first order decay best fit the data.^20,31^ Reported first order bulk decay coefficients from field and simulation studies range from 0.21 to 120 × 10^–3^ h^–1^ for free chlorine and 0.17–6.8 × 10^–3^ h^–1^ for chloramine decay.^20,26–31^ We found only one report in which second order chloramine decay was described for simulated drinking water.^32^ N^th^ order decay, where reaction order *n* is an optimized noninteger value, has been used to evaluate bulk chlorine decay in a few studies but never in premise plumbing.^33,34^

Water distribution system design and operation can influence biofilm growth and rates of pipe corrosion, which can increase chloramine decay rates far beyond those occurring in bulk water. In studies with reactive iron and cement in laboratory or full water distribution systems, first-order wall decay rate constants were at least 400% greater than decay constants in bulk water.^26,27,31,35^ Wall effects were less significant for plastics (PVC, uPVC, polyethylene), which do not corrode or support thick rust and biofilm layers.^31,35^

Nitrification is a common challenge in water mains, where it can accelerate chloramine decay. A critical threshold chloramine residual level has been described, below which ammonia-oxidizing bacteria (AOB) regrowth exceeds inactivation in a DWDS.^10,36–38^ Exceeding this threshold inhibits nitrification. In DWDSs, it is generally understood that elevated seasonal temperatures can also increase chloramine decay due to nitrification,^15,39,40^ and one study described the effect of seasonal temperature shifts on the critical chloramine threshold for nitrification control.^38^

Recent attempts have been made to extend research on chlorine and chloramine decay kinetics from main DWDSs into premise plumbing systems to better understand effects on *Legionella*, *Mycobacteria*, and other opportunistic pathogens (OPs).^3,13,16,41^ Given the presence of more reactive materials such as copper,^18,19,42^ higher surface-area-to-volume ratios, stagnation, elevated water age, higher levels of microbial growth, and warm water,^7,15,43–45^ it is not surprising that extremely high decay rates and nitrification have been reported in premise plumbing. Chloramine decay rate has also been found to have a positive correlation with biofilm age.^6^ Of the seven studies reporting a premise plumbing decay order, six simply assumed a first or pseudo first order disinfectant decay model,^3,22,46–49^ whereas Xu et al. found decay could occasionally be second order in the presence of organic matter.^13^ Premise plumbing and associated biofilms can also cause a rapid residual loss. One field study examining copper premise plumbing found first-order chloramine decay rates from water in the building to be 20–144 times higher than for the same water maintained in a glass container.^22^ Similarly, a study on premise plumbing pipe sections reported copper, galvanized iron, and PVC pipes were associated with first-order chlorine decay rate constants between 0.24 to 1.57 h^–1^,^49^ which are 7–131 times higher than constants reported for bulk water in other studies simulating water mains.^27,35^

Given the array of phenomena that can contribute to chloramine decay in premise plumbing, the net result is that in-building chloramine concentrations can often fall below the thresholds that have been established to control nitrification in DWDS water mains.^16,50^ There is relatively little research on how to control nitrification in buildings, since regulations on chloramine residuals, nitrate, and nitrite that could prompt such studies do not apply to stagnant water samples collected in buildings.^51^ It is understood that water age, Reynolds number, and other hydrodynamic factors influence chlorine decay rates and nitrification in wastewater treatment plants, reclaimed water distribution networks, and DWDSs,^17,35,48,52–57^ but analogous research is lacking for premise plumbing.

Here we examine the interplay between hydraulic design, water retention time (i.e., building water age), and temperature on nitrification and disinfectant decay kinetics in an at-scale plumbing rig with mature biofilms (4–6 years old) at influent chloramine levels of <0.2, 0.25, 0.5, 1.0, and 2.5 mg/L. Chloramine decay rates in glass jars without biofilm were compared to PEX pipes containing mature biofilms to shed light on decay kinetics under premise plumbing conditions. Overall, the study improves understanding of the kinetics of chloramine decay in premise plumbing, which in turn can help to improve OP control strategies.

## Methods

### At-Scale Premise Plumbing Rig Configuration and Operation

The at-scale pipe rig and its operation are described in detail by Busch et al. (2024).^58^ Briefly, 16 at-scale pipe legs were connected to the Blacksburg, VA chloraminated water supply naturally containing nitrifying bacteria.^59,60^ Between 0 to 100% of the water entering the pipes was filtered through in-line granular activated carbon (GAC) filters (Pentair, Golden Valley, MN) to reduce influent total chloramine to a targeted range from the ∼2.5 mg/L residual in the public water supply to <0.05 mg/L as Cl_2_. This water flowed to a manifold, where it was distributed to cold-water pipes or to a 19-gallon electric water heater for heating prior to distribution to hot-water pipes. There were a total of 8 cold and 8 hot water cross-linked polyethylene (PEX-B) water pipe legs, each 134-feet long (SharkBite, Atlanta, GA). A backflow preventer, ball valve, and check valves prevented cross contamination between the pipes. Water flow was controlled by automated timers (ChronTrol, San Diego, CA) and solenoid valves (ASCO Red Hat, St. Louis, MO). Either one or two sampling taps were located along the flow paths of each pipe segment to sample different water retention times (WRT) within the rig from 1 to 21.3 days (Figure 1).^58^ The pipes developed mature biofilms over the course of four years of operation before the research described herein. Prior studies from our group describe the most abundant phyla of mature biofilms in rig systems fed with Blacksburg tap water.^61,62^

**Figure 1:**
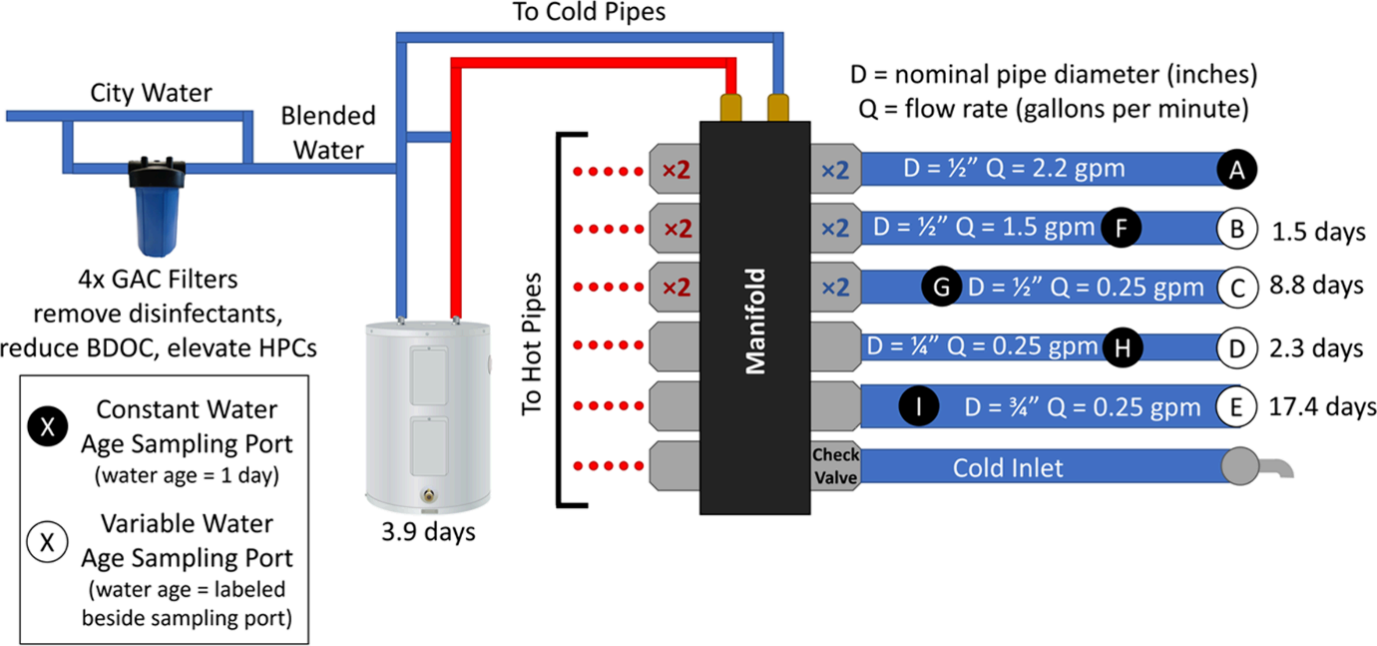
At-scale premise plumbing rig detailed in Busch et al.^58^ Identical cold and hot water pipe branches examine the effect of varying the pipe diameter, flow rate, and water retention time (WRT) on disinfectant residuals and opportunistic pathogen growth. The top three pipe segments A, B, and C are duplicated, resulting in a total of 16 cold and hot pipe branches. Sampling ports are installed at strategic distances along each pipe to reflect important water use and design scenarios of constant 1-day WRT (black circles) or at the end of the pipe with variable WRT (white circles).

### Experimental Design

Six phases of research were conducted to examine the effects of ambient temperature and influent chloramine levels (Table 1). GAC filtration produced bacterial growth representative of that which occurs in whole building filtration systems^63,64^ and also simulated the general effects of very high distribution system water age in terms of lower residuals, lower nutrients, and high cell counts.^65–67^ The proportion of municipal water mixed with GAC-filtered water was changed to achieve target residuals. Each pipe experienced flow for 35 s once daily. During this study, the water heater was set at 40 °C, which delivered water to the pipes at a temperature of 32–35 °C, a range that is ideal for OP growth. The apparatus was acclimated for at least one month at each target influent chloramine residual before sampling for chemical and biological constituents. Given that ambient room temperatures can range from 17 to 31 °C,^68^ the first phase of experiments was conducted at the lower end of this range (∼18 °C), whereas the remaining phases were conducted toward the middle (∼25 °C) (Table 1). Phase II was repeated twice to check the repeatability of the findings.

**Table 1:** Summary of the Experimental Phases and Corresponding Conditions

				Cold Water Influent (Municipal Water Supply)			Hot Water Influent (Water Heater)		
Phase	Dates	Average Room T (°C)	Average NH_2_Cl levels entering cold pipes and water heater (mg/L)^a^	T^b^ (°C)	pH^b^	DO^b^ (mg/L)	T^b^ (°C)	pH^b^	DO^b^(mg/L)
I	7/2021–1/2022	18	0.42	12.7	7.49	12.04	33.5	7.45	6.34
II	2/2022–12/2022	23	0.08	22.8 ± 0.10	7.29 ± 0.11	8.44 ± 0.15	32.4 ± 1.10	7.30 ± 0.01	6.40 ± 0.18
III	12/2022–3/2023	23	0.26	13.9	7.34	10.28	33.7	7.33	7.36
IV	3/2023–5/2023	25	0.46	18.2	7.15	9.34	35.0	7.41	7.04
V	5/2023–8/2023	25	0.94	23.8	7.04	8.35	34.4	7.45	6.34
VI	9/2023–10/2023	25	2.47	24.3	7.53	8.55	35.1	7.54	6.92

a Measured as total chlorine.

b Reported values are averaged across measurements made during the corresponding phase.

### Rig Sampling and Analysis

Over this 2.25-year experiment, more than 1,300 samples were collected and analyzed as follows.

#### Water Sample Collection

One or two sampling events of the cold water influent, water heater effluent (i.e., hot water pipe influent), and each sample tap on the pipe rig were conducted for each Phase. Pipe tap samples of 0.56-L volume were collected in polypropylene bottles after ≈24 h without flow. Pipe influent samples were collected after 10 min of flushing the blended water (cold water pipes) or from the water heater effluent after ≈1 min of flushing (hot water pipes).

#### Water Chemistry Analysis

Temperature, pH, dissolved oxygen (DO), disinfectant residual, total ammonia, nitrate, and nitrite were measured in each sample. Temperature and the pH were measured using a pH 150 m (Oakton Research, Vernon Hills, IL). DO was measured using a YSI ProSOLO ODO meter (YSI, Yellow Springs, OH). A DR3900 or DR5000 HACH spectrophotometer was used to quantify chloramine residual measured as total chlorine with DPD (Method 8167, HACH), total ammonia with Salicylate (Method 8155, HACH), and nitrite with Diazotization (Method 8507, HACH, Loveland, CO). Nitrate was quantified using inductively coupled plasma mass spectrometry (iCAP RQ ICP-MS; Thermo Fisher Scientific, Waltham, WA). Nitrification was measured by the conversion of total ammonia to nitrite or nitrate.

### Rig Disinfectant Decay

Chloramine during stagnation in each pipe was monitored at influent residuals of 0.5, 1.0, and ∼2.5 mg/L Cl_2_ after the rig had acclimated to each disinfectant dose for at least one month. Unexpectedly, disinfectant concentrations were always near or below the limit of detection at every tap after 24 h of stagnation (Figure S1). Therefore, it was only necessary to monitor decay rates in the cold water taps with a 1-day WRT (8 locations). On the days of sampling, these taps were flushed for 2–3× pipe volumes to ensure that chloramine at the taps was equal to the influent disinfectant level. Thereafter, stagnation was initiated, and disinfectant residuals were assessed hourly by collecting 15 mL aliquots from each pipe with time.

### Disinfectant Decay in Glass Jars

A complementary bench-scale experiment was conducted to characterize the kinetics of free chlorine and chloramine decay in water without plumbing materials or mature biofilms. For these tests, Blacksburg tap water was breakpoint chlorinated to reach 3 mg/L of free Cl_2_ after a 24-h hold time. This water was then aliquoted under three experimental conditions. In the first, the water was inoculated with 4% (v/v) GAC-treated water (≈182 total cells/mL) that was similar to the GAC-filtered water blended for the various influent conditions in the rig. In the second, in addition to inoculating with 4% v/v of GAC influent water, the water was dosed with ammonia and chlorine to achieve a residual of 6.84 mg/L chloramine as Cl_2_ (4:1 Cl_2_:N weight ratio). This dose was selected to challenge microbial growth, creating the least likely scenario for biological chloramine decay at the upper range of chloramine levels naturally present in the public water supply. In the third, chloramine was dosed in the same manner, but GAC influent water was not inoculated. Water from each condition was transferred into 15 250 mL clean, heat-sterilized borosilicate glass jars and incubated in the dark at 5, 19, 24, 30, and 39 °C in triplicate on orbital shakers rotating at 50 rpm. Every 2–3 days, free chlorine and monochloramine were measured along with total cell counts.

### Disinfectant Decay Modeling

Chloramine decay in the pipe rig was modeled by using two methods. The first was the linearized integrated rate law (LIRL) in which eq 1 is integrated into a linear form

$$\frac{C^{1-n}}{1-n}=-kt+\frac{C_{0}^{1-n}}{1-n}\;\mathrm{for}\;n\neq 1$$ 2

$$\ln C=-kt+\ln C_{0}\;\mathrm{for}\;n=1$$ 3

where *C*_0_ is the initial chloramine concentration (mg/L), and *k* is fixed as the inverse of the slope. LIRL modeling was performed in Excel.

Decay was also modeled by the nonlinear least-squares (NLS) Gauss–Newton algorithm in RStudio version 4.4.1 by solving eq 1 for chloramine:

$$C={\left(\left(n-1\right)kt+C_{0}^{1-n}\right)}^{1/1-n}\;\mathrm{for}\;n\neq 1$$ 4

5 $$C=C_{0}e^{-kt}\;\mathrm{for}\;n=1$$

For both methods, *n* was evaluated as an integer (i.e., 0, 1, or 2) or a noninteger value. Comparisons of the fit between the methods were made with residual standard error (RSE).

### Statistical Analysis

Statistical tests were performed in RStudio version 4.4.1. Data was checked for normality using the Shapiro-Wilk test and equal variance using the Bartlett and Levene’s tests. An ANOVA followed by the Tukey HSD test was used to compare the means under bulk water disinfectant decay conditions. An unpaired student’s *t* test was conducted to compare means. When normality and equal variance criteria were not met, an unpaired Wilcoxon Rank Sum test was performed. An alpha value of 0.05 was applied for all tests.

## Results

### Physicochemical Parameters

#### Undetectable Levels of Chloramine After 24-h Water Retention Time in Pipe Rig

Even with influent chloramine levels up to ∼2.5 mg/L, chloramine residuals were near or below detection after 24 h of stagnation in all cold water pipes (Figure S1). This was the case at both ambient temperatures tested (18 or 25 °C). Samples from the water heater, which was held at an average temperature of 39.6 °C (range 36.9–42.2 °C) (Figure S2), did not exceed 0.05 mg/L chloramine until the influent chloramine concentration reached 1 mg/L or higher. When the influent chloramine to the water heater was boosted to ∼2.5 mg/L, effluent chloramine still never rose above 0.10 mg/L. After the water from the water heater stagnated in hot water distal pipes for 24 h, the chloramine residual was only measured >0.05 mg/L on three occasions (data not shown).

#### Temperature and pH in the Pipe Rig

Cold water entering the rig varied seasonally, ranging from 12.7 °C–24.3 °C over the course of the 2+ year experiment (Table 1). Despite a thermostat set point of 40 °C and WRT of about 4 days, temperatures leaving the water heater were only 31.3–35.1 °C. Pipes in cold water distal lines always warmed to room temperature within 1 h stagnation, whereas hot water distal lines cooled to room temperature within 2.7 h of stagnation. As a result, temperatures in the portion of hot and cold pipes before the 1-day WRT taps were essentially room temperature for at least 21/24 h each day, and always at room temperature for portions of the pipes with higher WRT. Thus, all pipes were maintained at a temperature suitable for OP and OP host growth (i.e., >20 °C) at least 96% of the time during Phases II–VI.^69^

The pH of the blended cold water influent ranged from 7.04 to 7.53 throughout the experiment, while water heater samples ranged from 7.29 to 7.54. The pH typically decreased by a few tenths of a unit as it passed through the pipes.

### Disinfectant Decay in Sterilized Glass Containers without Biofilm

The lack of detectable disinfectant residuals in the pipe rig after 24 h stagnation prompted complementary experiments investigating the effect of temperature on disinfectant decay in inert glass jars and whether biological activity associated with natural drinking water flora (i.e., GAC inoculum) had any effect. The conventional wisdom is that chlorine and chloramine decay rates increase as temperature increases.^70,71^ This trend was confirmed for free chlorine in glass jars with the same local town water and GAC inoculum, in which the decay rates ranked as follows: 5 °C > 19 or 24 °C > 30 or 39 °C (Figure 2a). The expected trend was also confirmed for monochloramine in the town water without GAC inoculum, where after 20 days there was a 20.6% decrease in disinfectant at 5 °C compared to a 91.1% drop at 39 °C (Figure 2b).

**Figure 2:**
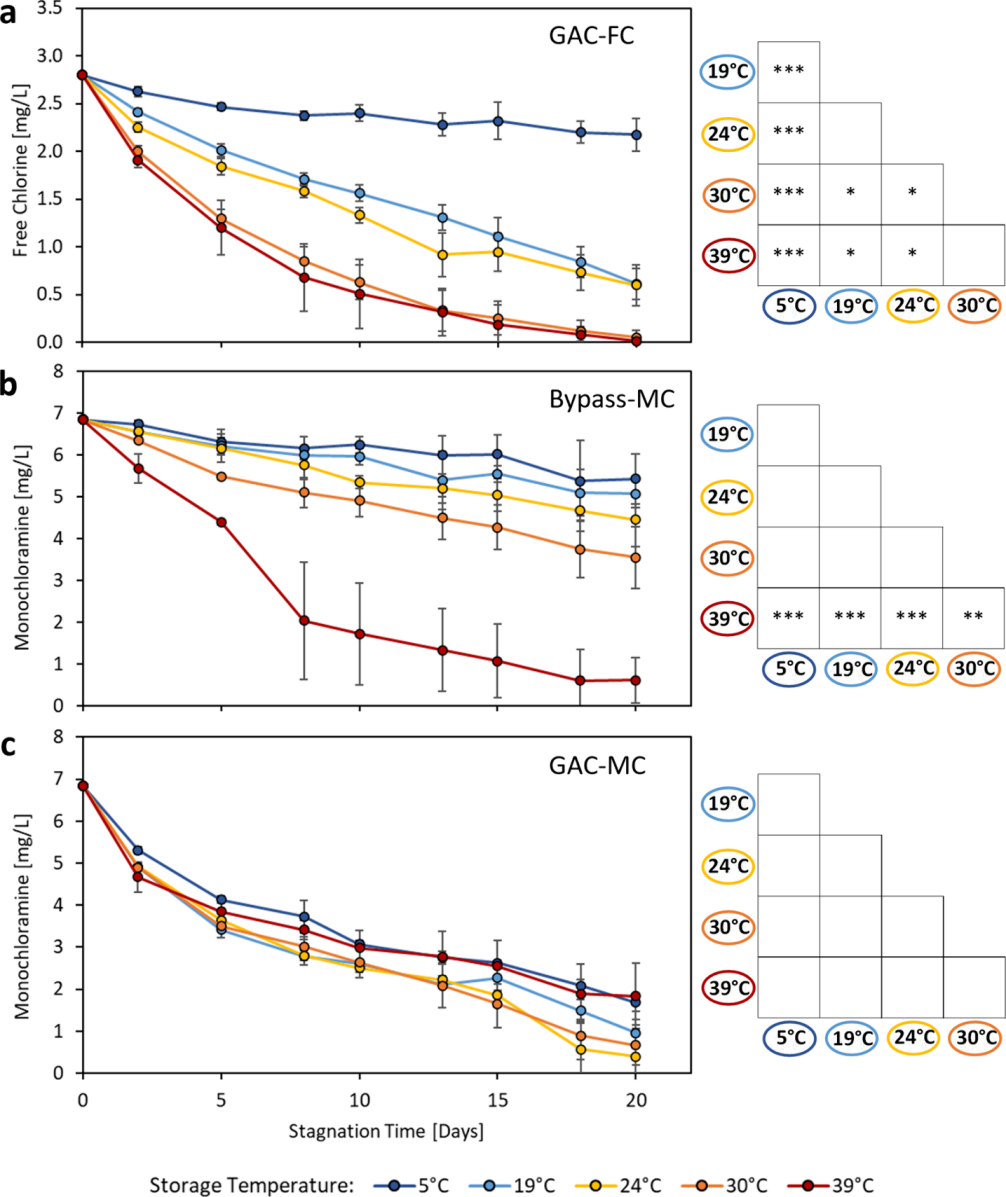
Disinfectant decay curves in bulk tap water containing (a) GAC-FC, free chlorine residual, and GAC inoculum; (b) Bypass-MC, chloramine residual without GAC inoculum; and (c) GAC-MC, chloramine residual with GAC inoculum. The GAC inoculum was effluent water from an aged GAC filter added at 4% v/v with the intention of adding the same natural drinking water flora containing nitrifiers that enters the premise plumbing rig. ANOVA post hoc TukeyHSD test results to the right of each graph show the level of difference between mean residuals (*n* = 3) for each temperature after 20-day stagnation with p-value significance thresholds of 0 to <0.001 ‘***’, 0.001 to <0.01 ‘**’, 0.01 to 0.05 ‘*’, and >0.05 ‘ ’.

However, the conventional wisdom did not hold in jars with monochloramine that received a GAC inoculum. Though not statistically significant (ANOVA post hoc Tukey HSD, p-values = 0.105 and 0.224), jars with the inoculum had an 18–29% *slower* rate of decay at 39 °C, when compared to cooler water at 19 or 24 °C room temperatures (Figure 2c). When this test was repeated, no chloramine residual remained at 25 or 30 °C after 30 days, but jars held at 37 °C contained significantly higher average chloramine of 0.33 mg/L (n = 9, p = 0.0153; data not shown). This finding aligns with reports that the optimal growth temperature for nitrifying bacteria in drinking and surface water is typically 20–30 °C.^38,72–74^

#### Disinfectant Decay Kinetics in Bulk Water

Disinfectant decay data from the glass jar experiments were fit with zero-, first-, or second order decay rate coefficients using the LIRL method (Table S1). The assumption of first order decay produced R^2^ values > 0.9 in 27/45 jars. R^2^ values for the data were only above 0.9 in 24/45 jars when assuming zero order and 19/45 jars when assuming second order. Additional details on bulk water decay kinetics are available in SI 1 and Table S2.

### Disinfectant Decay Kinetics in the Pipe Rig

The general approach used in the jar tests was then applied to chloramine decay kinetics in the pipe rig water for 1-day WRT taps over the range of pipe diameters and hydraulic conditions that were held stagnant following flushing. Sampling on the time scale of minutes to hours of stagnation revealed that chloramine was almost always completely undetectable within 8 h (Figure S3). In fact, at chloramine levels 0.5, 1.0, and ∼2.5 mg/L an average of 92% of the chloramine was lost in 4 h.

#### Optimizing Decay Kinetics

First or second order kinetics did not consistently provide a good fit to the data using either the LIRL (Figure 3a) or NLS (Figure 3b) approaches, requiring use of nonintegral (i.e., n^th^) reaction order. For instance, the 3/4 in. 0.25 gpm pipe full decay data at an influent concentration of 2.5 mg/L for 6.5 months was best fit with an LIRL n of 1.36 (RSE = 0.085 mg/L) and NLS n of 1.55 (RSE = 0.032 mg/L). First and second order decay models increased error on the fit for this pipe by about 440% for both the linear LIRL and NLS methods compared to n^th^ order models. Additional details are provided in Table S3.

**Figure 3:**
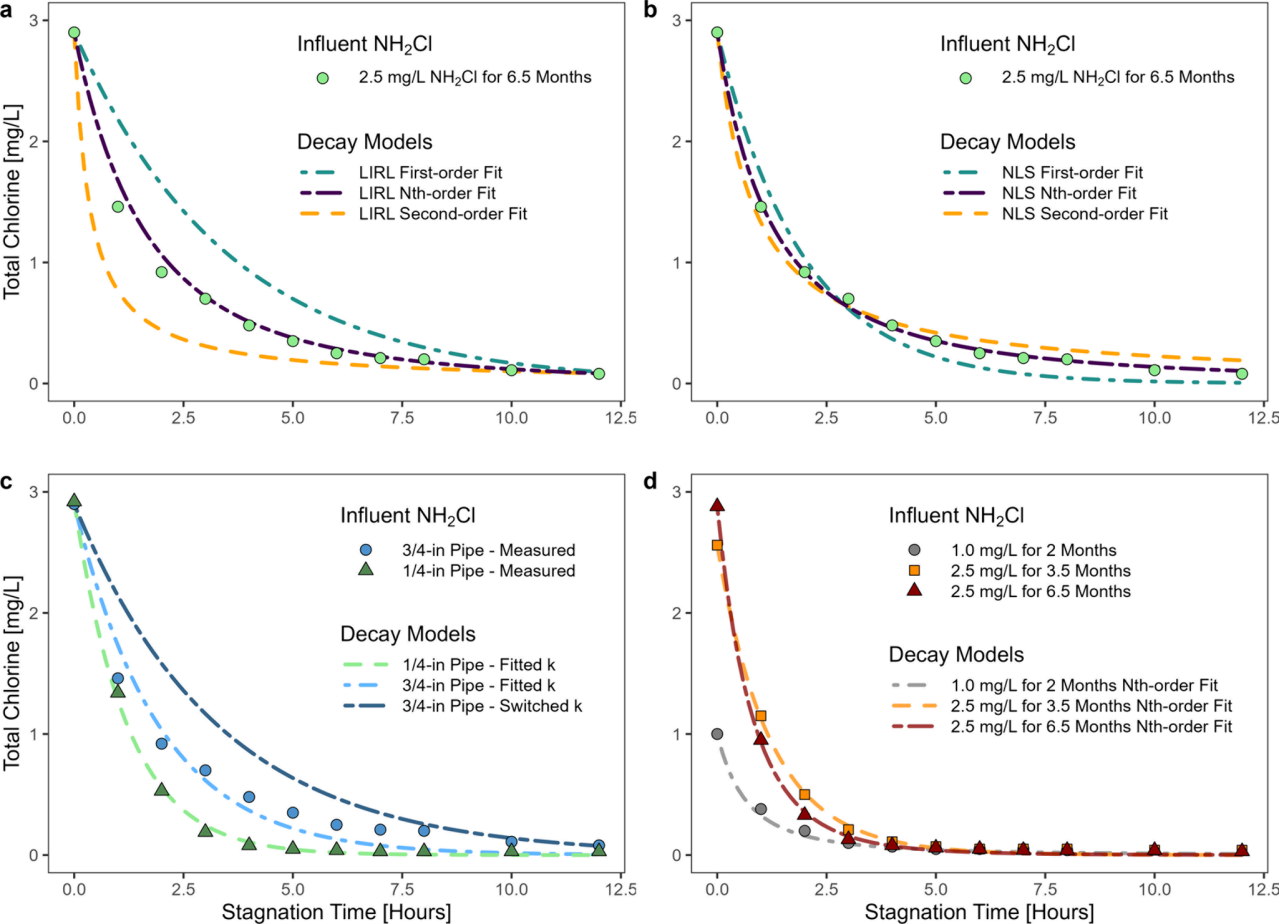
First, n^th^, and second order models of the 3/4” 0.25 gpm tap using the a) linear integrated rate law (LIRL) and b) nonlinear least-squares (NLS) methods; c) EPANET first order wall decay model fitted to 1/4” and 3/4” 0.25 gpm taps and with pipe specific and nonpipe specific decay coefficients; d) Decay curves for the 1/2” pipe at 0.25 gpm, Rep 2 tap at influent concentrations of 1.0 mg/L for 2 months, 2.5 mg/L for 3.5 months, and 2.5 mg/L for 6.5 months mg/L Cl_2_ fitted with n^th^ order NLS models. All measurements are from cold, 1-day WRT taps at a room temperature of 25 °C and measured as total chlorine. Models were fitted to begin at the initial disinfectant concentration measured at each tap and evaluate the full 12 h decay curve.

#### Decay Coefficients Were Pipe Specific

Optimum chloramine decay coefficients and reaction orders changed from tap to tap. Thus, the EPANET first order wall reaction model, which assumes that decay coefficients in a system can be modeled at varying pipe diameters (Equation S1), did not apply across pipes for chloramine decay in this study. For example, the model could find a k that reasonably fit data for the 1/4 in. pipe (RSE = 0.034 mg/L) (Figure 3c). With somewhat less accuracy, the first order model could also fit data for the 3/4-in. pipe (RSE = 0.139 mg/L). However, when the k found to optimize the 1/4-in. pipe was applied to the 3/4-in. pipe (or vice versa), the model did not fit very well (RSE = 0.359 mg/L). Hence, no single decay model could precisely predict disinfectant decay observed at different taps.

#### Variable Pipe Decay Kinetics

The overall response of the pipes to sustained high influent residuals of ∼2.5 mg/L chloramine was of interest to understand the long-term implications of in-building disinfection. We hypothesized that ∼2.5 mg/L would exceed the threshold required to control nitrification and other microbial activities, gradually leading to higher chloramine residuals after stagnation. Contrary to expectation, the time for chloramine to decay from the starting dose to 0.05 mg/L *decreased* with continued exposure to the higher chloramine dose in 2 of 8 cases, remained consistent in 4 of 8 cases, and increased in 2 of 8 cases (Figure S4), suggesting that the pipe biofilm could sometimes become more resistant toward disinfection.^75,76^ In cases where decay time decreased with exposure, as demonstrated in a 1/2 in., 0.25 gpm pipe (Figure 3d), chloramine rapidly decayed within the first hour despite exposure to a high disinfectant dose for 6.5 months.

Interestingly, in the one set of duplicate pipes at the highest flow velocity, there was also a divergence in the disinfectant decay profile. That is, for one of the duplicate pipes, the disinfectant concentration decreased to a trace residual after 10 h, whereas the other pipe required 14 h to reach the same concentration. The pipe with a higher rate of nitrification, as evidenced by 0.13 mg/L higher nitrate after 14 h stagnation, produced the faster chloramine decay rate.

In our preliminary study on this rig after it had been operating for 1.5 years at ≈25 °C, chloramine in the cold pipes decayed from 1.0 to 0.1 mg/L in 2–24 h.^58^ After biofilm in these pipes had aged for 4 more years, the time for chloramine to decay from 1.0 to 0.1 mg/L in cold water pipes at the same temperature was unchanged for the highest velocity pipes but decreased by 5.5–8.8× in other pipes (Figure S5). Plastic plumbing materials, including PEX, are considered relatively inert with disinfectant demand associated with carbon leaching typically reported to fade within months.^35,77^ As such, the increased rate of chloramine loss in the pipe rig could be attributed to microbial reactions.

#### Decay Orders Shifted for Pipes

When disinfectant decay data for the cold 1-day WRT taps were forced to best fit either first or second order using the LIRL method, three pipes that were best fit by second order decay shifted to best fit by first order decay during different phases of testing (Table S4). Decay constants for the three pipes with first order kinetics ranged between 0.26 and 0.41 h^–1^. R^2^ values were >0.9 for 33% of the first order fits and for 79% of the second order fits. Changes in optimal decay order and decay coefficient occurred using both the LIRL and NLS methods (Table 2). To make the comparisons on changing n and k over time on a similar basis for influent residuals of 1–2.5 mg/L, we fit only data below 1 mg/L. Overall, n ranged from 0.88 to 2.74 depending on the influent chloramine level and exposure time, hydraulics, and modeling method. The greatest change in reaction order occurred in pipes with the highest surface-area-to-volume ratio or highest flow velocity. In these three pipes the optimal n decreased by 0.88–0.95 after 6.5 months at an influent chloramine concentration of 2.5 mg/L (Table 2 pipes 1/2, 2.2, R1 LIRL Method; 1/2, 2.2, R2 LIRL Method; and 1/4, 0.25 NLS Method). Chloramine residuals below approximately 0.1 mg/L may be organic chloramine and have little disinfection efficiency,^78^ so the optimal decay order and coefficient were also calculated with residual values below 0.1 mg/L removed (Table S5). Even with a smaller decay order range (0.94–1.58), decay was not effectively modeled as either first or second order throughout all of the pipes.

**Table 2:** Optimal Decay Orders and Reaction Coefficients in Pipes at 25 °C Ambient Temperature^a^ ^,^ ^g^

	1.0 mg/L Influent NH_2_Cl for 2 Months			2.5 mg/L Influent NH_2_Cl for 3.5 Months^c^			2.5 mg/L Influent NH_2_Cl for 6.5 Months^c^		
Pipe Diameter (in), Flow Rate (gpm), Replicate^d^	Optimal Decay Order (n)	RSE^b^ (mg/L)	Reaction Coefficient (k)^e^	Optimal Decay Order (n)	RSE^b^ (mg/L)	Reaction Coefficient (k)^e^	Optimal Decay Order (n)	RSE^b^ (mg/L)	Reaction Coefficient (k)^e^
	**Linear Integrated Rate Law**								
1/2, 2.2, R1	2.16	0.049	6.03	1.37	0.186	0.75	1.28	0.160	0.67
1/2, 2.2, R2	2.15	0.056	5.91	1.18	0.051	0.54	1.20	0.195	0.44
1/2, 1.5, R1	2.51	0.058	15.61	2.34	0.229	10.25	2.74	0.221	34.35
1/2, 1.5, R2	2.26	0.075	6.23	2.31	0.311	8.37	2.27	0.307	9.25
1/2, 0.25, R1	1.91	0.052	2.85	1.50	0.035	0.90	1.80	0.192	2.22
1/2, 0.25, R2	2.07	0.061	3.71	2.38	0.373	7.54	2.41	0.290	8.91
3/4, 0.25	1.97	0.055	1.98	1.33	0.144	0.41	1.28	0.142	0.37
1/4, 0.25	2.26	0.022	4.80	2.61	0.404	22.56	2.06	0.437	5.25
	**Nonlinear Least Squares**								
1/2, 2.2, R1	1.47	0.016	1.56	1.06	0.012	0.49	1.04	0.020	0.52
1/2, 2.2, R2	1.39	0.018	1.31	1.13	0.007	0.50	0.97	0.016	0.35
1/2, 1.5, R1	1.44	0.018	1.56	1.15	0.029	1.16	1.23	0.022	1.33
1/2, 1.5, R2	1.58	0.011	1.60	0.98	0.043	0.98	0.96	0.036	1.11
1/2, 0.25, R1	1.34	0.015	1.11	1.50	0.015	0.83	1.31	0.020	1.05
1/2, 0.25, R2	1.49	0.012	1.26	1.05	0.031	0.80	1.16	0.030	1.05
3/4, 0.25	1.50	0.014	0.89	1.56	0.021	0.52	1.49	0.031	0.44
1/4, 0.25^f^	1.82	0.013	2.51	–	–	–	0.88	0.029	0.86

a Initial disinfectant concentrations (*C*_0_) are set to the initial total chlorine concentration measured at the tap after flushing 2–3× pipe volumes.

b RSE = Residual standard error.

c Adjusted to start decay near 1.0 mg/L and decay for approximately 8 h.

d The ability to replicate pipe conditions was limited by plumbing manifold ports.

e Units for reaction coefficient (*k*) vary based on the optimal decay order (*n*) to have units of L^*n*‑1^/(mg^*n*‑1^ × h).

f Instance where model did not converge is denoted with “–”.

g R^2^ values for the Linear Integrated Rate Law method are in Table S6.

A similar analysis could not be conducted for the hot water pipes because the vast majority of the chloramine decay occurred in the water heater. Chloramine levels leaving the water heater were below detection for Phases I–IV and rose slightly to 0.07 mg/L during Phase V and 0.10 mg/L during Phase VI. Levels of total chlorine at the hot taps were typically undetectable (average = 0.01 mg/L) after 1 day.

### Nitrification in the Pipe Rig

#### Cold Water Nitrification Was Typically Complete in 1 Day

Water passing through the GAC filter was partially nitrified (about 40% conversion of ammonia to nitrate).^79,80^ Thus, total ammonia delivered to the pipes increased up to 0.56 mg/L as N as the percentage of GAC bypass water and chloramine residual increased. Both steps of nitrification—free ammonia to nitrite conversion and nitrite to nitrate conversion—were complete in cold water pipes after a 1-day WRT (Figure 4). The only two exceptions were at the highest chloramine dose in the 0.25 gpm 3/4 in. and 1/2 in. diameter pipes, where nitrite to nitrate conversion was 68–85% complete after 1-day WRT. Nitrification was complete at all taps with a WRT higher than 1 day.

**Figure 4:**
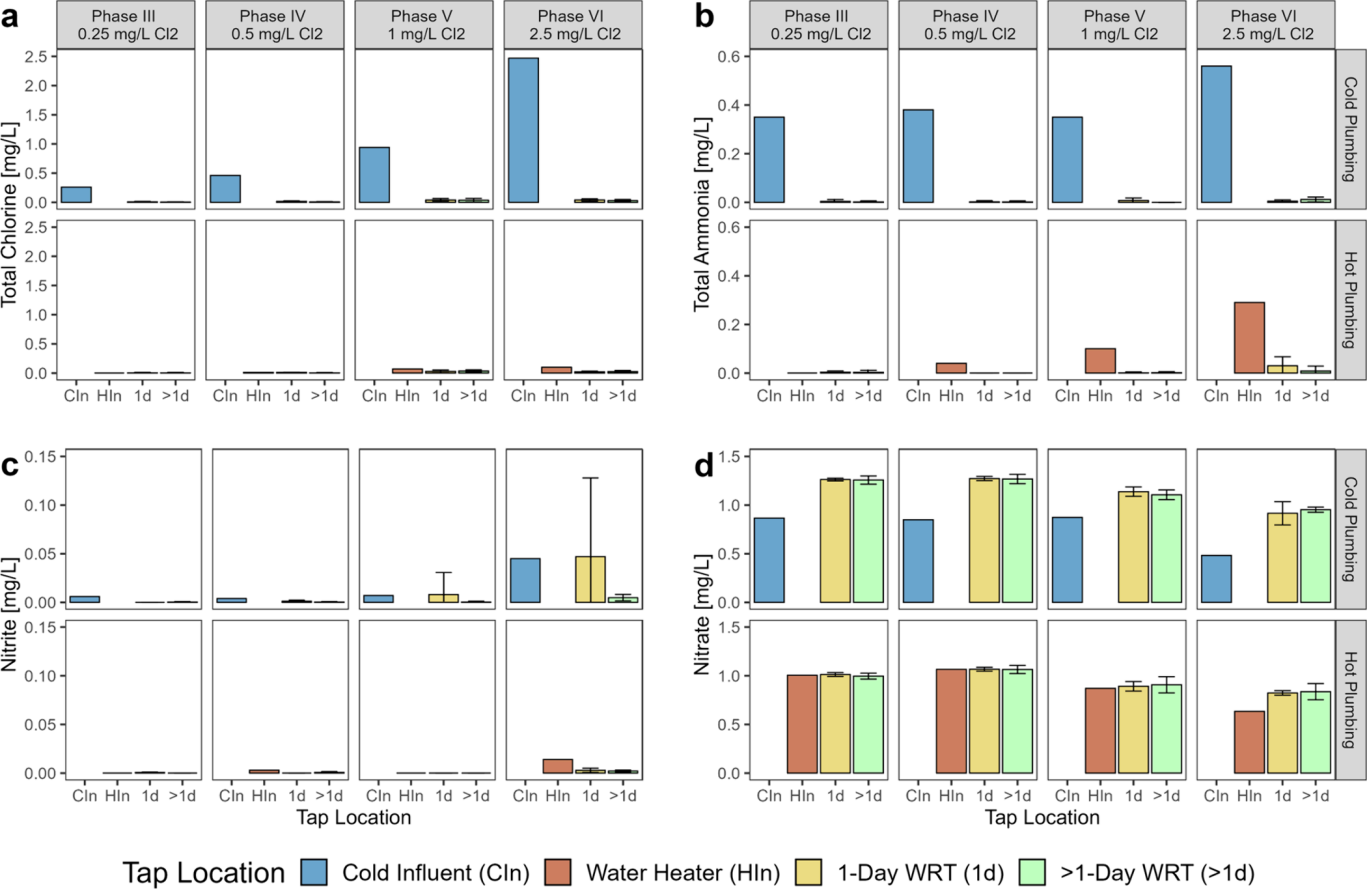
Indicators of nitrification in rig cold and hot plumbing as (a) total chlorine, (b) total ammonia, (c) nitrite, and (d) nitrate at a room temperature of 23–25 °C. Error bars represent standard deviation for 1-day (*n* = 8 for cold and hot separately) and above 1-day (*n* = 6 for cold and hot separately) taps.

#### Hot Water Nitrification and Denitrification Occurred in the Water Heater

During Phases III and IV, total ammonia and nitrite in the water heater were at or near zero, indicating that nitrification was likely completed in the tank (Figure 4). As chloramine dose increased to 2.5 mg/L, the percentage of influent ammonia passing through the tank also increased to 52%. In the hot plumbing, total ammonia was completely converted after 1-day, except during Phase VI. Nitrification occurred slower in the water heater compared to the pipes, presumably due to its lower surface-area-to-volume ratio and a water heater set point above the optimum nitrifier growth temperature. This was consistent with the jar experiments, in which the GAC filtrate inoculum caused faster decay at room temperatures of 19–25 °C compared to temperatures near the water heater set point (37–39 °C). Evidence of some denitrification, which can occur under anoxic conditions,^53^ was also observed in the water heater during all phases, illustrated by a 0.26 mg/L loss of total nitrogen sampled from >1-day hot water taps compared to >1-day cold taps during Phase III (Figure 4), and lower nitrate in hot pipes than in the cold water influent in 21% of samples during Phase V.

## Discussion

### Bulk Water Disinfectant Decay Kinetics

Temperature is known to be a master variable affecting all chemical and biological chloramine decay reactions; however, this study revealed that the temperature does not always influence kinetics as expected. The Arrhenius relationship has been reported to effectively model the effect of temperature on first and second (bilinear) order chlorine decay coefficients.^81–83^ Nonetheless, this study reveals a potentially important exception to the rule in chloraminated systems with a GAC inoculum, as lower temperatures of 19–30 °C produced faster decay than at 37–39 °C. First order decay coefficients for monochloramine without a GAC inoculum increased with temperature as expected, implicating that microorganisms in the GAC inoculum were the cause of the difference. Free chlorine decay rates were not affected by GAC inoculum (Figure S6), which further implicates microbes that specifically affect chloramine, i.e., nitrifiers. We hypothesize that the lower temperatures might be more optimal for nitrifiers that were present when compared to 37–39 °C, which caused faster nitrification and correspondingly faster loss of chloramine at 19–30 °C. Fisher and colleagues posited that cases where deviation from the Arrhenius relationship occurs could be due to low initial chlorine concentration (∼0.2 to 0.4 mg/L),^81^ but the present work further suggests that it could be due to microbial activity, even at very high initial chloramine concentrations. The inability for an Arrhenius equation to describe the complex process of bacterial growth, including AOB, at suboptimal and above suboptimal growth temperatures was recognized by Ratkowsky et al. and by Sarker et al.^38,84^ We show here that this deviation from an Arrhenius relationship also can apply to chloramine decay in drinking water of premise plumbing, which can have a diverse microbiome.

### Applicability of Decay Models to Premise Plumbing

EPANET was designed for DWDSs, and prior studies highlighted its capability to model bulk chlorine decay in drinking water distribution systems as zero, first, second (bilinear), two-reactant second (bilinear), and n^th^ order.^85,86^ A prior study on bulk chlorine decay as a function of WRT in a distribution system found that water continually flowing through a large pipe had a similar decay rate as water sitting in a glass bottle,^28^ but this is clearly not the case for premise plumbing as demonstrated herein.

The large deviation from bulk water decay behavior is important because EPANET and similar models are now being widely applied as tools to model premise plumbing. It has been assumed that chlorine will exhibit first order decay kinetics in premise plumbing without any supporting data for this assumption.^3,22,46–49^ Adapting EPANET and other models to premise plumbing will be challenging because bulk and wall reactions will be highly variable from one PEX pipe to another PEX pipe in the same water and premise plumbing system, as demonstrated herein.

The findings of this study are consistent with those of a prior field study with copper pipe, where decay from the pipe wall was 20–144 times higher than bulk water reactions and variable from tap to tap.^22^ In the study conducted herein with 4–6 year old biofilms at 25 °C ambient temperature with PEX, chloramine decay time from about 1 to 0.05 mg/L decreased in the order bypass bulk water in glass jars (6884 h) ≫ GAC bulk water in glass jars (583.5 h) > high flow rate pipes (6.8 h) ≈ low flow rate pipes (5.8 h). Thus, the pipe wall and biofilm effect described herein can result in one, two, or even 3 orders of magnitude greater decay rates than the bulk water. Almost as importantly, best fit decay reaction orders varied dramatically and even changed with different influent chloramine levels in the exact same pipe at different times. An analysis of the bulk water and biofilm microbial community composition is in progress to identify which taxa are the largest contributors to changes in chloramine loss and nitrification at different phases of the study. Nonetheless, this study demonstrates that simply assuming decay constants, or first or second order decay reactions, could lead to dramatic errors in estimating chloramine residuals at the tap. Two prior studies on chloramine decay in DWDSs determined decay was better fit by first order kinetics in bulk water and pipe segments when compared to zero or second order kinetics.^20,31^ The study herein demonstrates that describing chloramine decay in complex premise plumbing systems will sometimes require consideration of nonintegral reaction orders. Decay optimization models such as mixed-order and parallel first order, which have previously improved on n^th^ order fit for bulk water chlorine decay data,^33,34^ may also be potential candidates for improving disinfectant decay predictions in premise plumbing. To our surprise, in this study designed to test worst case stagnation, hydraulics did not influence chloramine decay, perhaps because the pipes were stagnant 99.96% of the time (Figure S7). Under other flow scenarios beyond those covered herein, which warrant further investigation, we expect that hydraulics will significantly impact sediment deposition or removal, chloramine decay, and disinfection of biofilms. In addition to hydraulic variability, future studies can also elucidate the impact of factors such as influent water chemistry and pipe materials on disinfectant decay.

Clearly, more research is needed on chlorine and chloramine decay in stagnant pipes along the lines of research conducted herein before such systems can be reasonably modeled. This builds on recent warnings that AWWA standards C651 and C652 – for disinfection of water mains and storage facilities – should not be assumed to apply in premise plumbing systems.^87–89^

### Factors Associated with Premise Plumbing Can Intensify Nitrification

Conventional wisdom indicates that frequent flushing, a higher disinfectant residual, and lower temperatures should control nitrification, but this work shows that the critical level of chloramine may not be achievable for taps that are frequently stagnant when nitrification is rapid. Results from this study showed complete nitrification in stagnant cold pipes was almost always complete within 1 day at all but the highest chloramine dose. The notion of the inability to maintain sufficient chloramine levels in premise plumbing is further supported by prior studies which demonstrated that flushing several times per day was insufficient to suppress nitrification^16,42^ even at 4 mg/L Cl_2_ every 8 h in a premise plumbing system with PVC, copper, lead, and galvanized pipes.^16^ Beyond nitrification, heterotrophic bacteria and microbial products such as proteins can also contribute to chloramine decay and warrant consideration when assessing disinfectant decay in buildings.^90^

In the hot water plumbing, we speculate that nitrification rates were limited due to a warmer than ideal nitrifier growth temperature in the water heater and much higher competition for nutrients from heterotrophic bacteria, which readily grew in the tank.^58,91^ Lower nitrate in the hot plumbing is also attributed to denitrification, indicating that parts of the water heater are probably anoxic.^53^ These findings challenge conventional wisdom regarding the temperature dependency of chloramine decay. Based on the findings of this study, strategies used to mitigate nitrification in premise plumbing are strongly advised to consider the complexity of building water systems and the propensity for residual loss.

## Conclusions

This investigation of chloramine decay kinetics and nitrification within an at-scale premise plumbing rig and complementary glass jar experiments led to the following key observations:

- Chloraminated bulk water inoculated with natural drinking water flora containing nitrifiers had a more persistent residual at ∼37 °C, compared to lower ambient temperatures tested in the jar experiments (19–30 °C). This is consistent with nitrifier activity being reduced at higher temperatures, thus enabling longer persistence of disinfectant than at lower temperatures.
- Nitrification was virtually complete in the cold water pipes within 1 day in the low water use scenario tested herein. Similarly, a majority of nitrification occurred within the water heater at 40 °C before distribution to hot pipes.
- Chloramine decay did not follow first order kinetics for the vast majority of instances measured in the rig, demonstrating erroneous assumptions used in common modeling approaches. Decay rates can be one, two, or even 3 orders of magnitude higher in pipes with biofilm than for bulk water in glass jars.
- Even with the same influent water, PEX pipes produced wide variability in chloramine decay kinetics, including pipes that transitioned from second to first order decay in response to rising influent chloramine concentration or extended chloramine exposure.

This study reveals unexpected chloramine decay trends in premise plumbing systems. The findings have important implications for the application of chloramines as a control measure. One particularly consequential finding was that, for infrequently used taps in a system without recirculation, the room temperature setting can be more influential than the influent temperature or the water heater set point. Further, nitrifiers had a preferred growth niche at room temperature that can increase nitrification rates above those typically present in bulk water. It was also clear that assumptions and tools designed to model disinfectant decay in water mains did not directly translate to modeling the rapid and variable chloramine decay that can occur in building pipes. As such, models for disinfectant decay in premise plumbing will be best fit by using building specific decay kinetics.

## Supplementary Material



## Notes

Chloramine residual at rig taps before and after daily flush; Zero, first, and second order coefficients of determination and reaction coefficients for disinfectant decay in glass jars modeled with the linearized integrated rate law; Bulk Water Best Fit Decay Order; Optimal decay orders and reaction coefficients in bulk water modeled with the linearized integrated rate law; Temperature profile in water heater; Chloramine decay profiles in cold pipes; Error associated with comparisons of rig chloramine decay data and models in Figure 3; EPANET wall reaction model; Chloramine decay time in cold pipes; Change in chloramine decay time with rig age; First and second order coefficients of determination and reaction coefficients for disinfectant decay at cold 1-day WRT taps modeled with the linearized integrated rate law; Optimal Decay Orders and Reaction Coefficients in Pipes at a 25 °C Ambient Temperature with Data Below 0.1 mg/L Cl2 Removed; Coefficients of determination for optimal decay orders using the linear integrated rate law method; First order decay constants Arrhenius equation relationship; Impact of Hydraulic Design Characteristics on Chloramine in Cold and Hot Water Pipes (PDF)
