# Supplementary material for: Complexity of Chloramine Decay Kinetics in Premise Plumbing
Source: ACS ES T Water. 2026 Jan 30;6(2):1305–16. doi: 10.1021/acsestwater.5c01339 (PMC12910582; doi:10.1021/acsestwater.5c01339)
Supplement: Supplementary file 1 [file ew5c01339_si_001.pdf]

Supplementary Information for

COMPLEXITY OF CHLORAMINE DECAY KINETICS IN PREMISE PLUMBING

Tolulope O. Odimayomi[1]*, Darel C. Snead[1], Amy Pruden[1], and Marc A. Edwards[1]

[1]Via Department of Civil and Environmental Engineering, Virginia Tech, Blacksburg, Virginia 24061, United States

*Email: todimayo@vt.edu

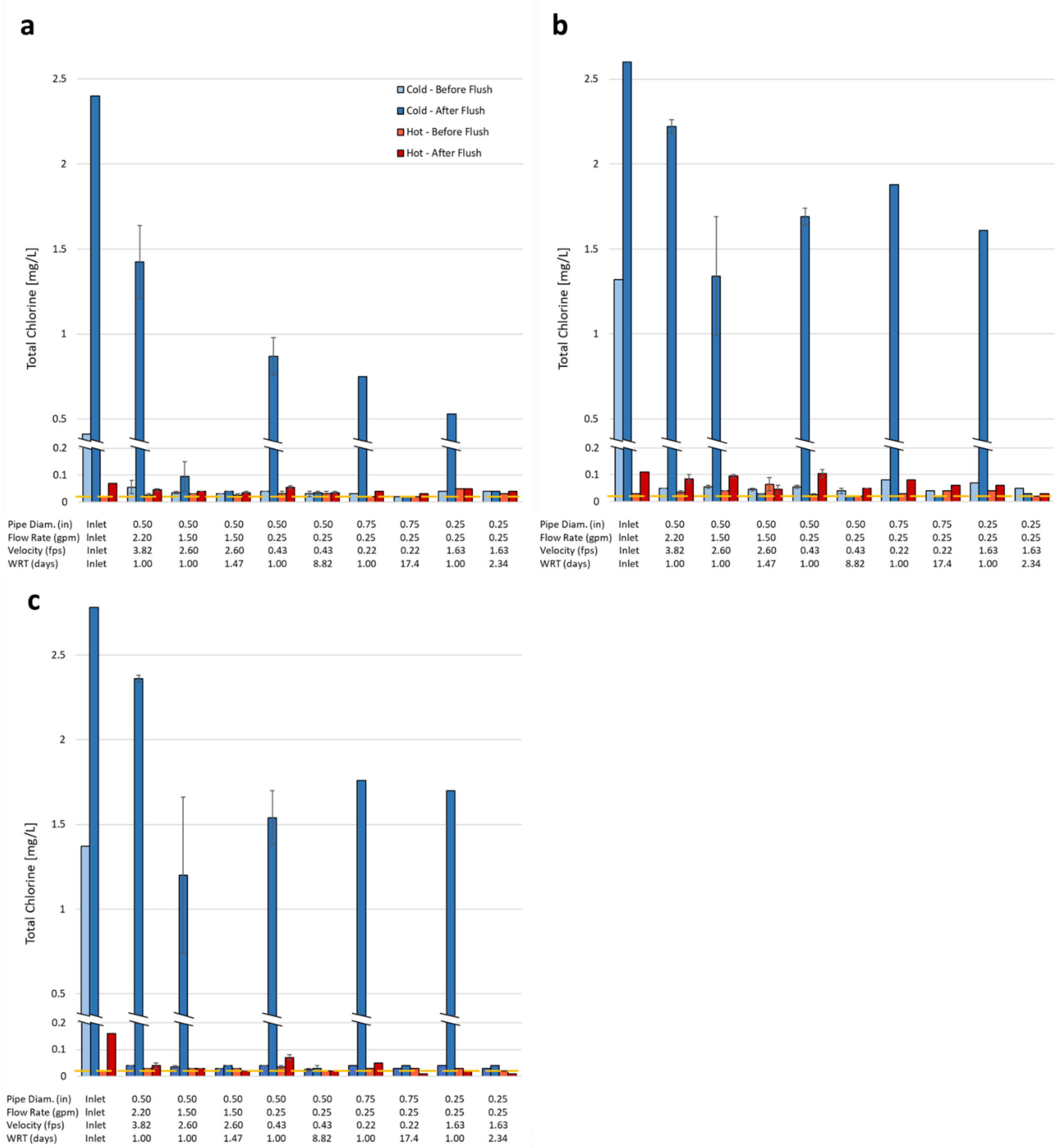

Figure S1. Chloramine residual at rig taps before and after daily flush. Total chlorine at the cold influent, water heater, and all distal taps a) 3, b)13, and c) 23 weeks after the switch to an influent disinfectant residual of 2.5 mg/L (Phase VI). Error bars show the standard deviation of duplicate taps. Horizontal dotted lines show the 0.02 mg/L limit of detection.

Table S1. Zero, first, and second order coefficients of determination and reaction coefficients for disinfectant decay in glass jars modeled with the linearized integrated rate law

	Free Chlorine Residual with GAC Inoculum (GAC-FC)		Chloramine Residual with GAC Inoculum (GAC-MC)		Chloramine Residual without GAC Inoculum (Bypass-MC)	
**Storage Temperature**	**R^2 Value**	**Reaction Coefficient k×10⁴***	**R^2 Value**	**Reaction Coefficient k×10⁴***	**R^2 Value**	**Reaction Coefficient k×10⁴***
	**Zero-Order Decay**					
5°C	0.896	11.43	0.909	93.50	0.923	28.70
19°C	0.986	42.72	0.855	100.2	0.969	36.61
24°C	0.948	43.16	0.922	117.1	0.987	49.13
30°C	0.876	52.59	0.916	112.7	0.976	65.56
39°C	0.846	52.02	0.848	85.97	0.878	130.4
	**First-Order Decay**					
5°C	0.918	4.741	0.977	26.29	0.903	4.947
19°C	0.970	29.64	0.936	34.89	0.964	6.350
24°C	0.986	32.38	**0.722**	88.64	0.993	8.991
30°C	0.962	92.07	0.848	63.72	0.991	13.61
39°C	0.975	103.6	0.960	26.08	0.956	87.77
	**Second-Order Decay**					
5°C	0.937	1.979	0.941	8.368	0.871	0.866
19°C	0.844	24.38	**0.761**	16.35	0.951	1.113
24°C	0.906	30.24	**0.326**	1502	0.989	1.678
30°C	**0.500**	931.6	**0.320**	748.7	0.978	2.957
39°C	**0.666**	1254	0.934	9.501	**0.593**	1338

*Units for reaction coefficients are [mg / (L × h)] for zero-order decay, [1/h] for first-order decay, and [L / (mg × h)] for second-order decay

Red: reaction order with R^2 <0.8

Yellow: two or three reaction orders having R^2 within 0.05 of each other

Green: reaction order with R^2 >0.9 and >0.05 greater than other orders

SI 1. Bulk Water Best Fit Decay Order

When disinfectant decay was very slow, as indicated by an average loss of <48% in 20 days (i.e., data for all 5°C jars and jars without GAC inoculum except 39°C), almost any reaction order could reasonably fit the experimental data (Table S1). The estimated optimal value for decay order (n) and decay coefficient (k) for bulk water were calculated to maximize the R^2 between measured disinfectant residuals and the nth order decay model (Table S1). Excluding the ambiguous cases with little decay, the best fit reaction order "n," ranged from 0.39-0.87 for free chlorine and 0.31-1.31 for chloramine when the GAC inoculum was present. There was no clear impact between temperature and best fit reaction order. The loss of disinfectant in the chloramine without GAC inoculum condition after 140 hours agreed with the reported auto-decomposition rate of chloramine at a similar pH, temperature, and alkalinity to Blacksburg tap water,[1,2] indicating that bulk water chloramine decay was likely driven by the instability of chloramine in water in the absence of the nitrifier containing inoculum.

Table S1. Optimal decay orders and reaction coefficients in bulk water modeled with the linearized integrated rate law

Water Type	Storage Temperature	Optimal Decay Order n	R^2 Value	Reaction Coefficient k $\times 10^4$ $[L^{n-1} / (mg^{n-1} \times h)]$*
Free Chlorine Residual with GAC Inoculum (GAC-FC)	5°C	6.40	0.977	0.05
	19°C	0.37	0.994	36.6
	24°C	0.86	0.987	33.3
	30°C	0.60	0.993	66.2
	39°C	0.64	0.980	73.7
Chloramine Residual with GAC Inoculum (GAC-MC)	5°C	1.17	0.979	21.5
	19°C	0.86	0.940	39.9
	24°C	0.32	0.952	90.1
	30°C	0.50	0.974	72.8
	39°C	1.31	0.968	18.7
Chloramine Residual without GAC Inoculum (Bypass-MC)	5°C	-1.54	0.933	440
	19°C	-0.38	0.970	71.8
	24°C	1.10	0.993	7.55
	30°C	1.06	0.991	12.4
	39°C	0.76	0.963	88.4

*Units for reaction coefficient (k) vary based on the optimal decay order (n)

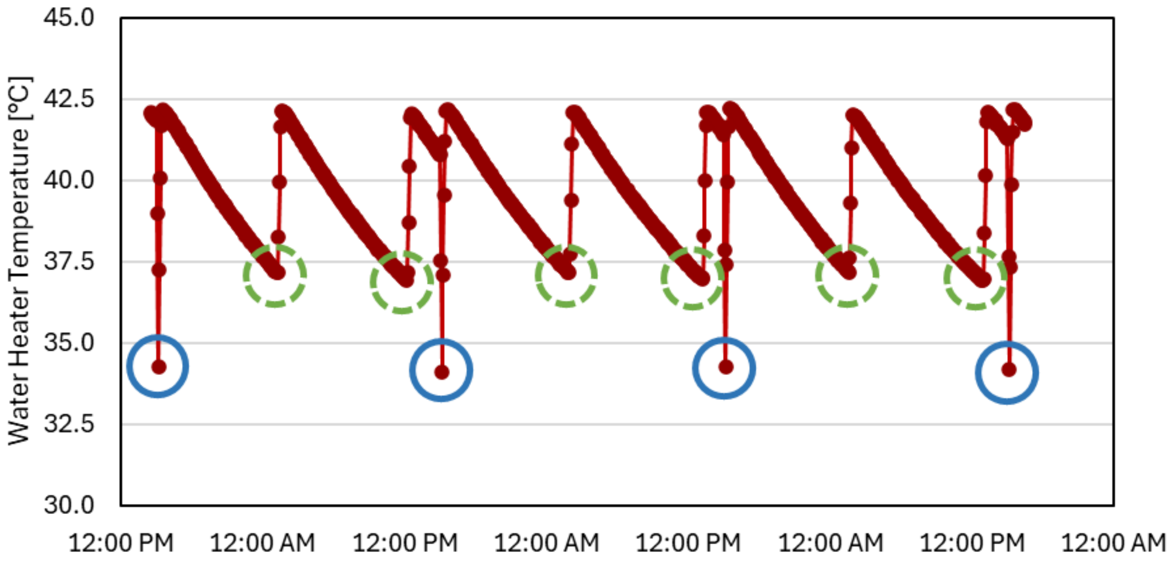

Figure S2. Temperature profile in water heater. Temperature profile over 3+ days in the 19-gallon water heater at a set point of 40°C. The heating element in the tank turned on when the water temperature fell below ~37°C (green dashed circles) or after the daily hot pipes flush cycle (blue solid circles).

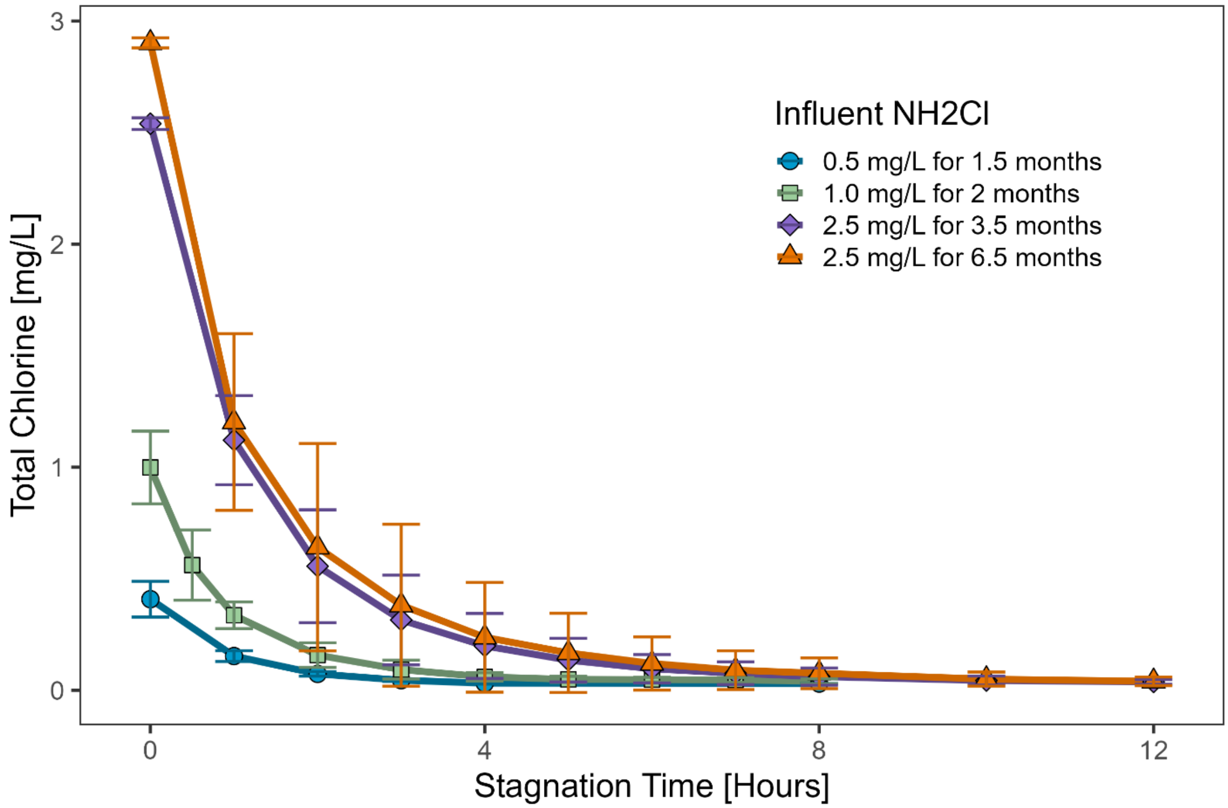

Figure S3. Chloramine decay profiles in cold pipes. Chloramine decay profiles for cold 1 day WRT taps measured as total chlorine (mg/L). Error bars show standard deviation for cold 1 day WRT taps (n=8). The legend specifies the duration of time taps received the influent chloramine concentration 1×/day for 35 seconds.

Table S3. Error associated with comparisons of rig chloramine decay data and models in Figure 3

Figure 3 Graph	Measured Data: Pipe Diameter (in), Flow Rate (gpm), Daily Flush Influent Chloramine (mg/L), Duration of Daily Flushing (months)	Model	RSE (mg/L)
a	3/4, 0.25, 2.5, 6.5	LIRL First-order	0.402
		LIRL n^{th}(1.36)-order	0.085
		LIRL Second-order	0.296
b	3/4, 0.25, 2.5, 6.5	NLS First-order	0.139
		NLS n^{th}(1.55)-order	0.032
		NLS Second-order	0.092
c	1/4, 0.25, 2.5, 6.5	EPANET Wall Decay First-order with 1/4" pipe fitted k	0.034
	3/4, 0.25, 2.5, 6.5	EPANET Wall Decay First-order with 3/4" pipe fitted k	0.139
		EPANET Wall Decay First-order with 1/4" pipe fitted k	0.359
d	1/2, 0.25, 1.0, 2	NLS n^{th}(1.49)-order	0.012
	1/2, 0.25, 2.5, 3.5	NLS n^{th}(1.05)-order	0.030
	1/2, 0.25, 2.5, 6.5	NLS n^{th}(1.16)-order	0.028

RSE = Residual standard error.

Equation S1. EPANET wall reaction model.[3]

$$R_w = (A/V)K_w C^n$$

R_w = rate of reaction at or near the pipe wall
(A/V) = surface-area-to-volume ratio within the pipe (equal to 4 divided by pipe diameter)
K_w = wall reaction rate coefficient
C = concentration in the bulk flow of the pipe
n = reaction order (either 0 or 1)

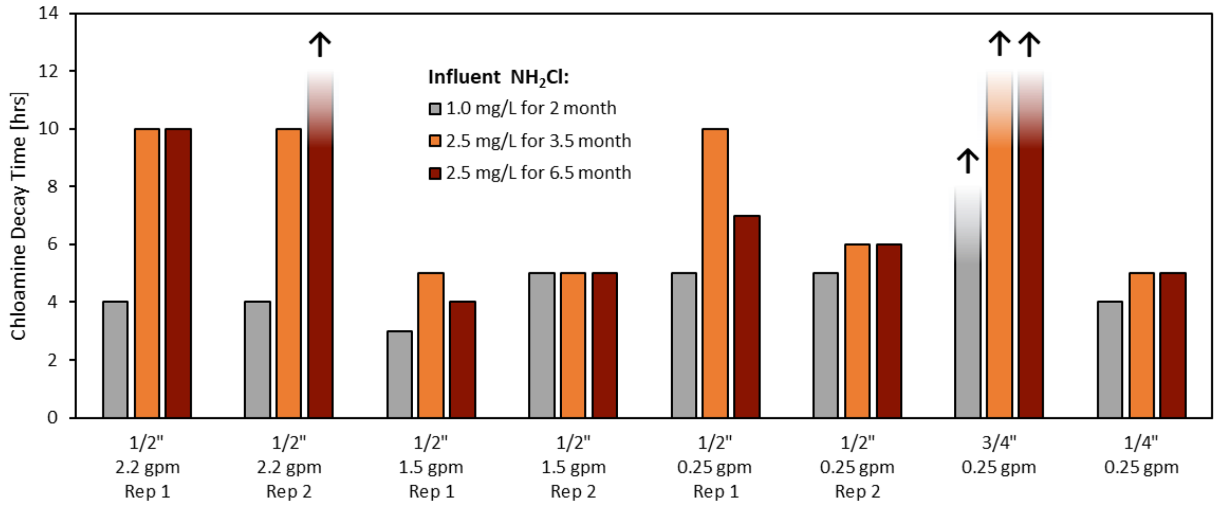

Figure S4. Chloramine decay time in cold pipes. Measured time for the chloramine residual to decay from the starting concentration to approximately 0.05 mg/L at cold, 1-day WRT taps with a room temperature of 25°C. Diagonal stripe fill: first order decay. Solid fill: second order decay or fit by both first and second order decay. Upward arrow: cases where a residual concentration of 0.05 mg/L was not reached by the final time point of 8 hours for the 1.0 mg/L influent chloramine condition or 12 hours for the 2.5 mg/L influent chloramine conditions.

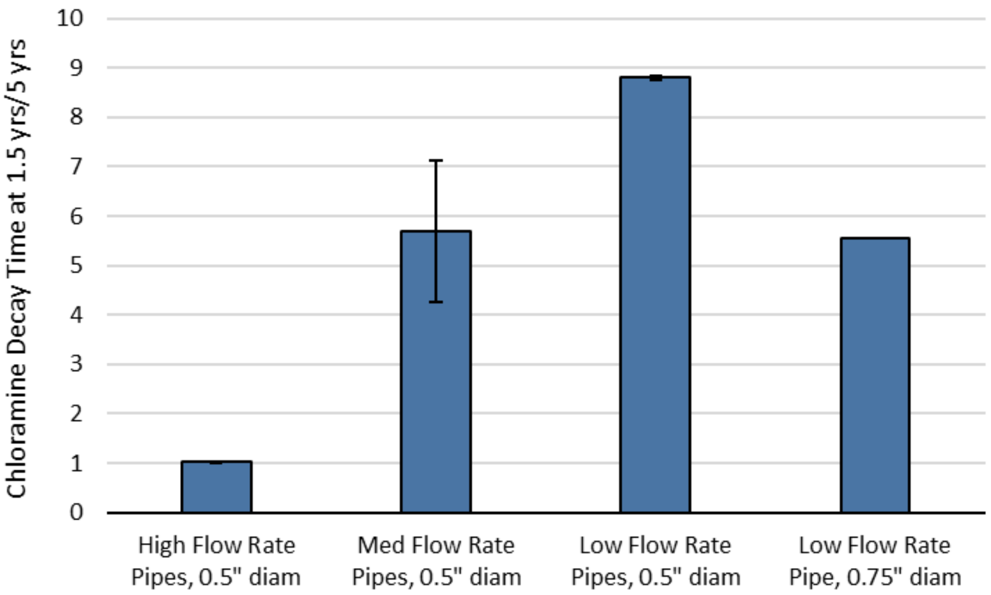

Figure S5. Change in chloramine decay time with rig age. Ratio of the time required for chloramine to decay from 1.0 to 0.1 mg/L in cold water 1-day WRT taps at a rig age of 1.5 years vs. 5 years. Low flow rate pipes: 0.25-0.39 gpm; Medium flow pipes: 1.37-1.5 gpm; High flow pipes: 1.99-2.2 gpm. Error bars represent the standard deviation for duplicate pipes.

Table S4. First and second order coefficients of determination and reaction coefficients for disinfectant decay at cold 1-day WRT taps modeled with the linearized integrated rate law

	1.0 mg/L Influent NH_2Cl for 2 months		2.5 mg/L Influent NH_2Cl for 3.5 months*		2.5 mg/L Influent NH_2Cl for 6.5 months*	
Pipe Diameter (in), Flow Rate (gpm), Replicate‡	R^2 Value	Reaction Coefficient k#	R^2 Value	Reaction Coefficient k#	R^2 Value	Reaction Coefficient k#
	First-Order Decay					
1/2, 2.2, R1	**0.802**	0.367	0.956	0.391	**0.981**	0.408
1/2, 2.2, R2	**0.814**	0.370	**0.992**	0.395	**0.991**	0.326
1/2, 1.5, R1	**0.746**	0.353	**0.761**	0.323	**0.710**	0.313
1/2, 1.5, R2	**0.823**	0.366	**0.779**	0.349	**0.726**	0.358
1/2, 0.25, R1	**0.831**	0.383	0.954	0.347	**0.878**	0.357
1/2, 0.25, R2	**0.850**	0.374	**0.811**	0.364	**0.759**	0.329
3/4, 0.25	0.903	0.331	**0.974**	0.277	**0.977**	0.262
1/4, 0.25	**0.716**	0.318	**0.731**	0.387	**0.800**	0.426
	Second-Order Decay					
1/2, 2.2, R1	**0.955**	3.936	0.942	2.623	0.908	2.890
1/2, 2.2, R2	**0.953**	3.968	**0.896**	2.877	0.922	1.748
1/2, 1.5, R1	**0.877§**	3.823	**0.950**	3.890	**0.890§**	3.964
1/2, 1.5, R2	**0.967**	3.290	**0.966**	3.666	**0.856§**	4.286
1/2, 0.25, R1	**0.909**	3.581	0.963	2.656	**0.981**	3.761
1/2, 0.25, R2	**0.956**	3.135	**0.938**	2.962	**0.914**	3.102
3/4, 0.25	**0.964**	2.110	0.928	1.057	0.931	0.939
1/4, 0.25	**0.827§**	2.606	**0.908**	4.057	**0.916**	4.475

*Adjusted to start decay near 1.0 mg/L
‡The ability to replicate pipe conditions was limited by plumbing manifold ports.
*Units for reaction coefficients are [1/h] for first-order decay and [L / (mg × h)] for second-order decay
Red: reaction order with R^2 <0.9
Red§: reaction order with highest R^2 but <0.9
Yellow: both reaction orders having R^2 within 0.025 of each other
Green: reaction order with R^2 >0.025 greater than other orders

Table S5. Optimal Decay Orders and Reaction Coefficients in Pipes at a 25°C Ambient Temperature with Data Below 0.1 mg/L Cl^2 Removed

	1.0 mg/L Influent NH_2Cl for 2 Months			2.5 mg/L Influent NH_2Cl for 3.5 Months*			2.5 mg/L Influent NH_2Cl for 6.5 Months*		
Pipe Diameter (in), Flow Rate (gpm), Replicate‡	Optimal Decay Order (n)	RSE (mg/L)	Reaction Coefficient (k)#	Optimal Decay Order (n)	RSE (mg/L)	Reaction Coefficient (k)#	Optimal Decay Order (n)	RSE (mg/L)	Reaction Coefficient (k)#
	**Linear Integrated Rate Law**								
1/2, 2.2, R1	--	--	--	1.02	0.031	0.48	1.27	0.176	0.65
1/2, 2.2, R2	--	--	--	1.04	0.064	0.46	1.03	0.071	0.37
1/2, 1.5, R1	--	--	--	--	--	--	--	--	--
1/2, 1.5, R2	--	--	--	--	--	--	--	--	--
1/2, 0.25, R1	--	--	--	1.26	0.176	0.65	1.60	0.203	1.50
1/2, 0.25, R2	1.23	0.016	1.00	1.15	0.134	0.93	--	--	--
3/4, 0.25	1.11	0.035	0.60	1.55	0.027	0.50	1.28	0.142	0.37
1/4, 0.25	--	--	--	1.18	0.356	1.34	--	--	--
	**Nonlinear Least Squares**								
1/2, 2.2, R1	--	--	--	1.06	0.007	0.49	1.02	0.018	0.52
1/2, 2.2, R2	--	--	--	1.13	0.007	0.50	0.94	0.012	0.35
1/2, 1.5, R1	--	--	--	--	--	--	--	--	--
1/2, 1.5, R2	--	--	--	--	--	--	--	--	--
1/2, 0.25, R1	--	--	--	1.54	0.016	0.82	1.25	0.018	1.06
1/2, 0.25, R2	1.37	0.008	1.15	0.98	0.011	0.81	--	--	--
3/4, 0.25	1.44	0.014	0.84	1.58	0.020	0.52	1.49	0.028	0.44
1/4, 0.25	--	--	--	0.97	0.012	0.81	--	--	--

Initial disinfectant concentrations (C_0) are set to the initial total chlorine concentration measured at the tap after flushing 2-3× pipe volumes.
RSE = Residual standard error
Conditions with <4 data points were insufficient for modelling and are denoted with "--"
*Adjusted to start decay near 1.0 mg/L and decay for approximately 8 hours.
‡The ability to replicate pipe conditions was limited by plumbing manifold ports.
#Units for reaction coefficient (k) vary based on the optimal decay order (n) to have units of L^{n-1} / (mg^{n-1} × h)

Table S6. Coefficients of determination for optimal decay orders using the linear integrated rate law method

	1.0 mg/L Influent NH_2Cl for 2 Months		2.5 mg/L Influent NH_2Cl for 3.5 Months*		2.5 mg/L Influent NH_2Cl for 6.5 Months*	
**Pipe Diameter (in), Flow Rate (gpm), Replicate‡**	**Optimal Decay Order (n)**	**R^2 Value**	**Optimal Decay Order (n)**	**R^2 Value**	**Optimal Decay Order (n)**	**R^2 Value**
1/2, 2.2, R1	2.16	0.957	1.37	0.972	1.28	0.999
1/2, 2.2, R2	2.15	0.955	1.18	0.999	1.20	0.998
1/2, 1.5, R1	2.51	0.891	2.34	0.959	2.74	0.917
1/2, 1.5, R2	2.26	0.971	2.31	0.974	2.27	0.859
1/2, 0.25, R1	1.91	0.909	1.50	0.997	1.80	0.986
1/2, 0.25, R2	2.07	0.956	2.38	0.945	2.41	0.923
3/4, 0.25	1.97	0.964	1.33	0.994	1.28	0.986
1/4, 0.25	2.26	0.830	2.61	0.928	2.06	0.916

*Adjusted to start decay near 1.0 mg/L

‡The ability to replicate pipe conditions was limited by plumbing manifold ports.

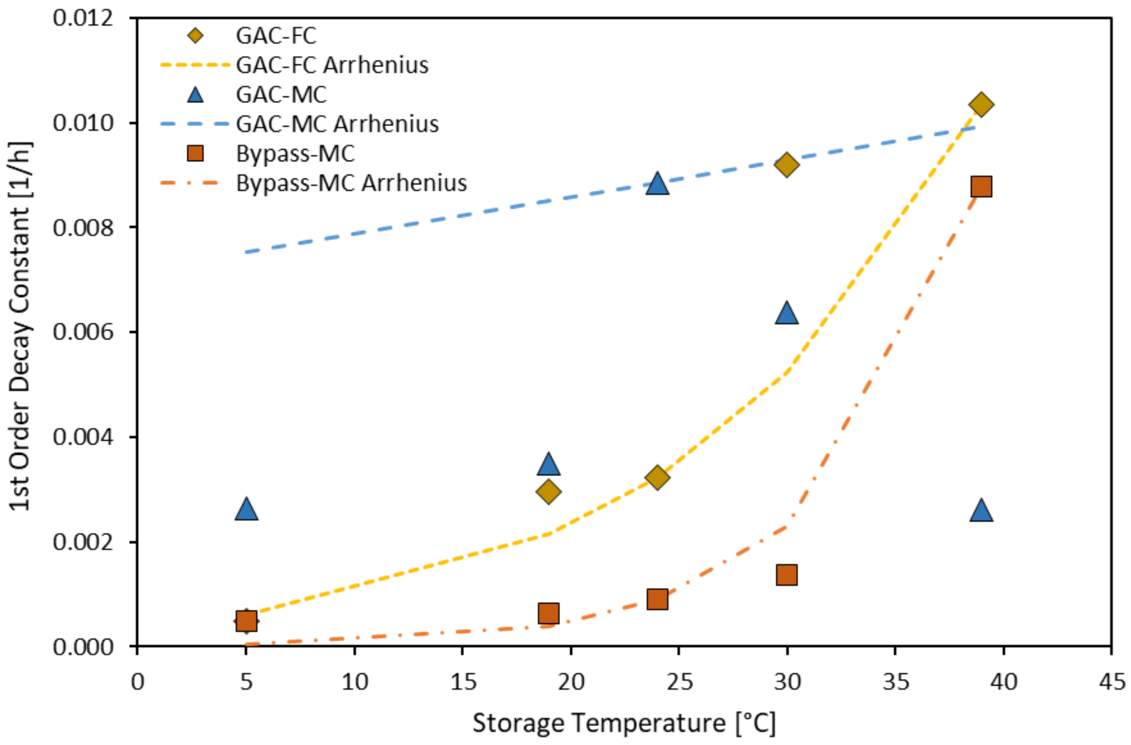

Figure S6. First order decay constants Arrhenius equation relationship. First order disinfectant decay constants (k) based on experimental data (points) and Arrhenius equation predictions (lines) for bulk water stored in glass jars for 20 days. GAC-FC: free chlorine residual and GAC inoculum, GAC-MC: chloramine residual with GAC inoculum, and Bypass-MC: chloramine residual without GAC inoculum.

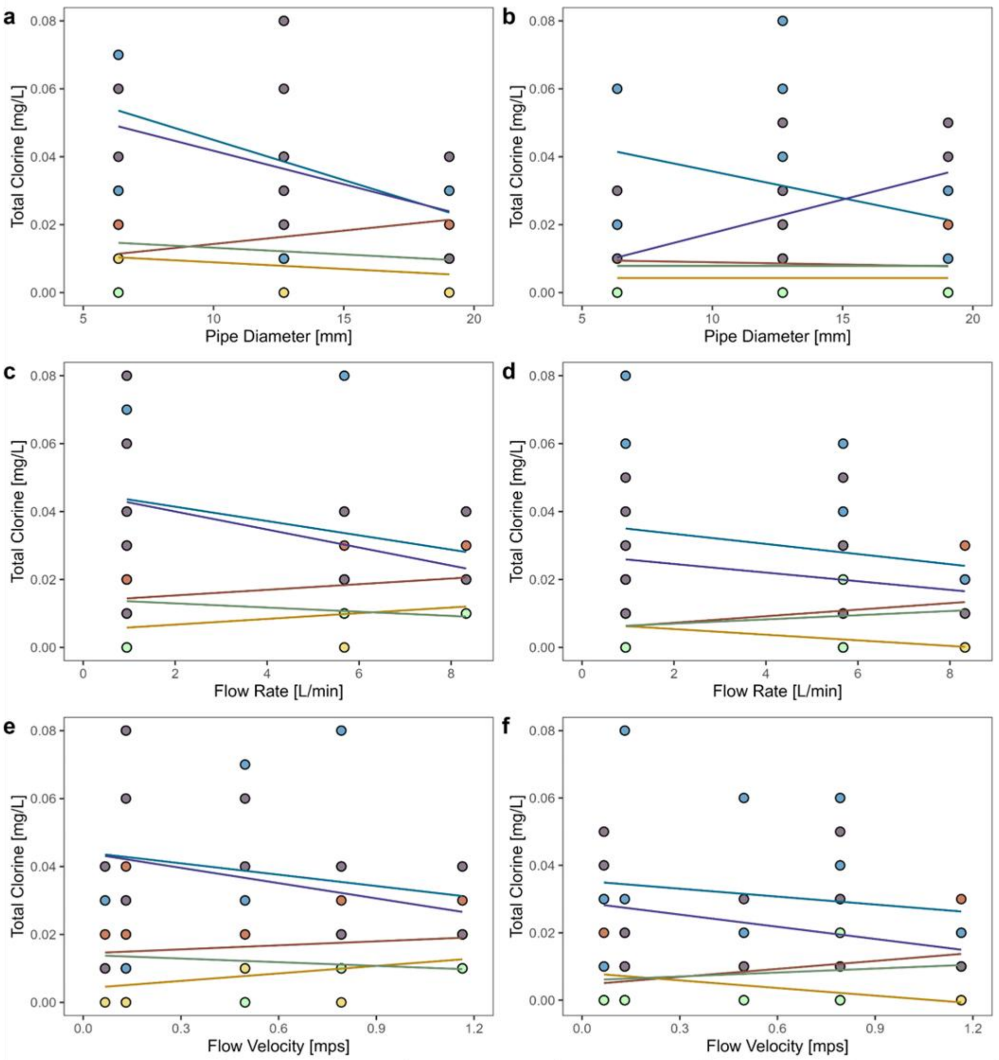

Influent NH_2Cl [mg/L]	<0.2 (Stages I & II)	0.25 (Stage III)	0.5 (Stage IV)	1.0 (Stage V)	2.5 (Stage VI)
a Pipe Diameter (Cold)					
b Pipe Diameter (Hot)					
c Flow Rate (Cold)					
d Flow Rate (Hot)					
e Flow Velocity (Cold)					
f Flow Velocity (Hot)	*				

Figure S7. Impact of Hydraulic Design Characteristics on Chloramine in Cold and Hot Water Pipes. Trends for chloramine (measured as total chlorine) after 24-hour stagnation in cold and hot water pipes as a function of pipe diameter (a and b), flow rate (c and d), and flow velocity (e and f). Linear model regression test results under the legend show significance by stage (n=14-42) with p-value significance thresholds of 0 to <0.001 '***', 0.001 to <0.01 '**', 0.01 to 0.05 '*', and >0.05 ' '.

Supplementary Information References

(1) Roy, R.; Sathasivan, A.; Kastl, G. Simplified Chemical Chloramine Decay Model for Water Distribution Systems. *Sci. Total Environ.* **2020**, *741*, 140410. https://doi.org/10.1016/j.scitotenv.2020.140410.
(2) Vikesland, P. J.; Ozekin, K.; Valentine, R. L. Monochloramine Decay in Model and Distribution System Waters. *Water Res.* **2001**, *35* (7), 1766–1776. https://doi.org/10.1016/S0043-1354(00)00406-1.
(3) Lew Rossman; Hyoungmin Woo; Michael Tryby; Feng Shang; Robert Janke; Terranna Haxton. *EPANET 2.2 User Manual*; EPA/600/R-20/133; U.S. Environmental Protection Agency: Washington, DC, 2020.
